# Exposure to Tobacco Smoke *in Utero* and Subsequent Plasma Lipids, ApoB, and CRP among Adult Women in the MoBa Cohort

**DOI:** 10.1289/ehp.1104563

**Published:** 2012-07-19

**Authors:** Lea A. Cupul-Uicab, Rolv Skjaerven, Kjell Haug, Gregory S. Travlos, Ralph E. Wilson, Merete Eggesbø, Jane A. Hoppin, Kristina W. Whitworth, Matthew P. Longnecker

**Affiliations:** 1 Epidemiology Branch, National Institute of Environmental Health Sciences, National Institutes of Health, Department of Health and Human Services, Research Triangle Park, North Carolina, USA; 2 Department of Public Health and Primary Health Care, University of Bergen, Bergen, Norway; 3 Medical Birth Registry of Norway, Norwegian Institute of Public Health, Bergen, Norway; 4 Cellular and Molecular Pathology Branch, National Institute of Environmental Health Sciences, National Institutes of Health, Department of Health and Human Services, Research Triangle Park, North Carolina, USA; 5 Department of Genes and Environment, Division of Epidemiology, Norwegian Institute of Public Health, Oslo, Norway

**Keywords:** clinical chemistry, C-reactive protein, metabolic syndrome, plasma lipids, prenatal exposure delayed effects, smoking, women

## Abstract

Background: Recent findings suggest that maternal smoking during pregnancy may play a role in the development of metabolic alterations in offspring during childhood. However, whether such exposure increases the risk of developing similar metabolic alterations during adulthood is uncertain.

Objective: We evaluated the association of *in utero* exposure to maternal tobacco smoke with plasma lipids, apolipoprotein B (apoB), and C-reactive protein (CRP) in adulthood.

Methods: The study was based on a subsample of the Norwegian Mother and Child Cohort Study (MoBa) and included 479 pregnant women with plasma lipids, apoB, and CRP measurements. Information on *in utero* exposure to tobacco smoke, personal smoking, and other factors were obtained from the women by a self-completed questionnaire at enrollment, at approximately 17 weeks of gestation.

Results: Women exposed to tobacco smoke *in utero* had higher triglycerides [10.7% higher; 95% confidence interval (CI): 3.9, 17.9] and lower high-density lipoprotein cholesterol (HDL) (–1.9 mg/dL; 95% CI: –4.3, 0.5) compared with unexposed women, after adjusting for age, physical activity, education, personal smoking, and current body mass index (BMI). Exposed women were also more likely to have triglycerides ≥ 200 mg/dL [adjusted odds ratio (aOR) = 2.5; 95% CI: 1.3, 5.1] and HDL < 50 mg/dL (aOR = 2.3; 95% CI: 1.1, 5.0). Low-density lipoprotein cholesterol, total cholesterol, and apoB were not associated with the exposure. CRP was increased among exposed women; however, after adjustment for BMI, the association was completely attenuated.

Conclusions: In this population, *in utero* exposure to tobacco smoke was associated with high triglycerides and low HDL in adulthood, 18–44 years after exposure.

Alterations in fetal programming in response to an adverse fetal environment may contribute to the development of metabolic syndrome (Gluckman and Hanson 2004). Components of the metabolic syndrome, such as elevated triglycerides and reduced high-density lipoprotein cholesterol (HDL), are known risk factors for cardiovascular disease and diabetes (Alberti et al. 2009). Recent findings from a longitudinal study suggest that maternal smoking during pregnancy may play a role in the development of metabolic alterations in offspring during childhood (Huang et al. 2007). However, whether *in utero* exposure to tobacco smoke is associated with metabolic alterations during adulthood is uncertain (Power et al. 2010) and the available epidemiologic data are insufficient. Previous studies, however, have reported an association of *in utero* exposure to tobacco smoke with cholesterol (Jaddoe et al. 2008; Wen et al. 2010) and carotid wall thickening during adulthood (Geerts et al. 2008). To our knowledge, other metabolic risk factors, such as elevated apolipoprotein B (apoB) and C-reactive protein (CRP), have not been studied in relation to *in utero* exposure to tobacco smoke. Elevated triglycerides and reduced HDL (i.e., atherogenic dyslipidemia) are usually accompanied by elevated apoB; CRP is a marker of inflammation that tends to be elevated in people with atherosclerotic conditions and metabolic syndrome (Grundy et al. 2004).

The purpose of the present study was to evaluate the association of *in utero* exposure to maternal tobacco smoke (i.e., exposure occurred 18–44 years earlier for women in the present analysis) with alterations in plasma lipids that are compatible with metabolic syndrome in adulthood. We also evaluated the association of *in utero* exposure to tobacco smoke with low-density lipoprotein cholesterol (LDL), total cholesterol, apoB, and CRP.

## Methods

This study was based on the Norwegian Mother and Child Cohort Study (MoBa), conducted by the Norwegian Institute of Public Health (Oslo, Norway) (Magnus et al. 2006). MoBa is a cohort based on 108,000 pregnancies from 90,700 women enrolled from 1999 to 2008. The majority of all pregnant women in Norway were invited to participate, and 38.5% of invited women participated in the study. Participants were recruited with a mailed invitation before a routine ultrasound examination offered to all pregnant women in Norway at 17–18 weeks of gestation (Norwegian Institute of Public Health 2007). The study was approved by the Regional Committee for Medical Research Ethics and the Norwegian Data Inspectorate (Oslo, Norway). Informed consent was provided by each participant. The present study is based on version 4.301 of the quality-assured data files released for research in December 2009. At enrollment, participants were asked to give a plasma specimen (median, 18 weeks of gestation; 25th and 75th percentiles, 17 and 19 weeks, respectively) and to complete a questionnaire about demographic characteristics, reproductive health, disease and medication history, lifestyle, and socioeconomic status. The present analysis was based on a subsample of 950 women whose plasma specimens were analyzed for lipids, apoB, and CRP for a case–base (Kupper et al. 1975) study on subfecundity (Whitworth et al. 2012). For the case–base study, eligibility was restricted to all participants enrolled in 2003 and 2004 who delivered a live-born child, provided a plasma specimen, and reported their time-to-pregnancy (*n* = 8,120). Cases (*n* = 400) were selected at random among eligible women who were subfecund (i.e., with a time-to-pregnancy of > 12 months; *n* = 839); the base sample (*n* = 550) was selected at random from all eligible women regardless of subfecundity status.

*Assessment of* in utero *tobacco smoke*. Women’s exposure to tobacco smoke *in utero* was ascertained on the baseline questionnaire. Women were asked “Did your mother smoke when she was pregnant with you?” Those who answered “yes” were classified as having been exposed to tobacco smoke *in utero*; those who responded “no” were considered unexposed. For women who participated with more than one pregnancy in the MoBa cohort, the consistency of answers across pregnancies was verified. In general, if the woman gave two different answers in two consecutive pregnancies (e.g., yes/no; no/yes; no/don’t know; yes/don’t know), the response was considered inconsistent, and the subject was excluded from the analysis. However, if the first answer was “Don’t know” and later she gave a different answer (yes or no), we used the latter under the assumption that the woman had asked her mother about her exposure *in utero*. Analyses of data from the MoBa cohort support the validity and reproducibility of self-reported *in utero* exposure to tobacco smoke among MoBa participants (Cupul-Uicab et al. 2011a, 2011b). Women were not asked about cigarette smoke exposure during childhood.

*Plasma measurements*. At enrollment, women provided a nonfasting blood specimen collected in EDTA tubes; the samples were shipped at ambient temperature from the collection site to Oslo where plasma was extracted and stored at –80° C (the majority of the samples were received the day after collection) (Ronningen et al. 2006). For the present study, we used the plasma specimens provided by the women at enrollment. Although some women participated with multiple pregnancies in MoBa, women could contribute with only one pregnancy in the present analysis. Triglycerides, total cholesterol, apoB, CRP, HDL, and LDL were determined in plasma specimens with an Olympus AU400e Clinical Chemistry Analyzer (Olympus America Inc., Irvin, TX, USA) using reagents for triglycerides and cholesterol from Beckman Coulter (Brea, CA, USA) and reagents for the others from Genzyme Diagnostics (Framingham, MA, USA). Quantitative measurement of LDL was done with a direct enzymatic method (N-geneous® LDL-ST cholesterol reagent). CRP was expressed in milligrams per liter and the other clinical chemistries were expressed in milligrams per deciliter. The within- and between-batch coefficients of variation (CV) of the analytical method were < 5% for all clinical chemistries, except for the between-batch CV for triglycerides (CV 6.3%).

*Statistical analysis*. For the present analysis, women from the case–base study (*n* = 950) were excluded if they had missing data on body mass index (BMI), education, or clinical chemistries (*n* = 8). In addition, women were excluded if, for *in utero* tobacco smoke, there was unknown exposure (*n* = 112) or inconsistent answers (*n* = 7) (see above). After all the exclusions, a total of 823 women were included in the analysis (base sample, *n* = 479).

The associations of *in utero* exposure to tobacco smoke with levels of triglycerides, HDL, LDL, total cholesterol, apoB, and CRP were assessed separately. The main analysis was restricted to women from the base sample (selected without regard to subfecundity status). We also conducted an analysis that included all women from the MoBa subsample using weighted linear regression to account for the case–base sampling (Richardson et al. 2007). The weights were the inverse of the sampling probabilities for the original case–base study. Triglycerides and CRP had skewed distributions and therefore were natural-logarithm transformed before modeling; their corresponding coefficients represent the percent difference in geometric mean values for the exposed compared with the unexposed groups. For some of the outcomes, we also assessed whether clinically relevant alterations in levels were associated with *in utero* exposure to tobacco smoke using logistic regression. We used previously recommended cut points to define high triglycerides (≥ 200 mg/dL), low HDL (< 50 mg/dL for females), high total cholesterol (≥ 240 mg/dL), and high CRP (≥ 8 mg/L) levels [National Cholesterol Education Program (NCEP) 2001; Pitiphat et al. 2005].

All models included *in utero* tobacco smoke as the main exposure and were adjusted for woman’s age (years), education (≤ high school vs. > high school), and physical activity [times a week that the woman was engaged in activities such as brisk walking, running/jogging/orienteering, bicycling, gymnastics, aerobics, dancing, skiing, or swimming (none, < 4, or ≥ 4 times a week)]. We use directed acyclic graphs (DAGs) to select this set of *a priori* variables (Greenland et al. 1999). Although the minimal sufficient set of variables to adjust for confounding did not include education, we choose to adjust for each participant’s education as a proxy indicator of her mother’s education and socioeconomic status [see Supplemental Material, Figure S1 (http://dx.doi.org/10.1289/ehp.1104563)]. Participants’ individual annual income was not selected *a priori* because of its correlation with education (Spearman *r* = 0.43, *p* < 0.01) and because the exposure and outcomes had stronger correlations with education than with income. Information on the education, socioeconomic status, and lifestyle habits of participants’ mothers was not ascertained in MoBa. Additional variables that were associated with at least one of the outcomes in bivariate analyses (*p* ≤ 0.20) were assessed as potential confounders [i.e., income, parity, and alcohol consumption (no, yes)] using the change in estimate method, starting with all variables in the models with deletion of one by one in a stepwise manner (Greenland 1989). None of the tested variables caused a change ≥ 10% in the coefficient for *in utero* smoking, thus we did not adjust for them. Participants’ personal smoking, BMI [weight (in kilograms) ÷ height (in meters squared)], and birth weight (in kilograms) were potential intermediate variables that were included in selected models. BMI was entered as a continuous variable in the models because the results were comparable to those obtained using four categories. The effect of adjusting for birth weight was evaluated among the subset of women born in 1967 or later [when the Medical Birth Registry of Norway (Bergen, Norway) was established] with available birth weight [88.8% of 752 (89% of 446 for the base sample)]. Multiplicative interactions of *in utero* exposure to tobacco smoke (yes/no) with personal smoking (yes/no), BMI, and birth weight were tested in linear regression models but are not presented because the *p*-values for all interaction terms were > 0.15. We also estimated associations of *in utero* exposure to tobacco smoke (yes/no) with the outcomes in the absence or presence of overweight and obesity (BMI < 25 and ≥ 25 kg/m^2^, respectively) to assess departures from additive effects (Hosmer and Lemeshow 1992). However, numbers were too sparse to conduct similar analyses for smoking and birth weight.

We conducted a number of sensitivity analyses to assess the robustness of our results. We added the participant’s individual income to models in addition to education because adjusting for both variables might be a more effective means of reducing confounding due to socioeconomic disadvantages throughout the participant’s life. We also estimated associations adjusted for age and physical activity only, the minimal sufficient set of covariates based on the DAG. Because lipid levels can vary throughout pregnancy (Vahratian et al. 2010) and plasma specimens were not provided at the same week of gestation for all women, the models were further adjusted for gestational week at blood draw.

In additional sensitivity analysis, multiple imputation by chained equations (van Buuren et al. 1999) was performed in the complete sample (i.e., 950 subjects from the original case–base study) to impute values for any variable with missing data. For the imputation procedure, we included all clinical chemistries (triglycerides and CRP entered as natural logarithm), *in utero* exposure to tobacco smoke, all characteristics listed in Table 1, and BMI before pregnancy, weight and gestational age at birth, year of birth, year of enrollment, and age of the participant’s mother at delivery. A total of 10 imputed data sets were generated using 20 cycles per imputation (van Buuren et al. 1999), and analyses were repeated using the imputed data. All analyses were done using Stata (Stata Statistical Software, release 10.1; StataCorp, College Station, TX, USA).

**Table 1:** Characteristics of women from the MoBa base sample at enrollment according to self-reported *in utero* exposure to tobacco smoke.

Characteristic	*n* (%)		*p*-Value^a^
All women	479	(28.0)
Age (years)	0.07
< 25	43	(27.9)
25–29	183	(34.4)
30–34	179	(21.8)
≥ 35	74	(27.0)
Education	< 0.01
< High school	32	(37.5)
High school	141	(39.0)
College	214	(22.9)
> College	92	(19.6)
Incomeb ($US)	0.07
< 30,847	119	(36.1)
30,847–46,269	200	(27.5)
46,270–61,693	115	(24.3)
> 61,693	45	(17.8)
Parity	0.71
0	207	(26.1)
1	186	(29.0)
≥ 2	86	(30.2)
BMI (kg/m2)	< 0.01
< 25.0	253	(22.9)
25.0 to < 30.0	161	(28.6)
≥ 30.0	65	(46.2)
Smoking (cigarettes/day)	0.02
Nonsmoker	378	(25.7)
< 10	87	(33.3)
≥ 10	14	(57.1)
Alcohol drinking	0.77
No (nondrinker)	362	(27.3)
Yes (drinker)	73	(31.5)
Missing	44	(27.3)
Physical activity (times/week)	0.07
None	99	(38.4)
< 4.0	261	(26.1)
4.0 to < 7.5	89	(24.7)
≥ 7.5	30	(20.0)
ap-Values are from Pearson’s chi-square test comparing exposed and unexposed across categories of each variable. bIndividual annual income.					

## Results

The prevalence of *in utero* exposure to tobacco smoke among women in the base sample was > 35% among those with less than a college education, a lower income, a BMI ≥ 30, who smoked more as adults, and who reported no physical activity (Table 1). Exposure prevalences according to participant characteristics were similar among all 823 women (data not shown). Among women from the base sample, 7.9% had high triglycerides, 6.9% had low HDL, 20.3% had high cholesterol, and 22.1% had high CRP, with similar prevalences among subfecund MoBa participants [see Supplemental Material, Table S1 (http://dx.doi.org/10.1289/ehp.1104563)]. Compared to subfecund women, those from the base sample tended to be younger, and a higher proportion of them were college educated, had a previous pregnancy, had a BMI of < 25, and were nonsmokers (see Supplemental Material, Table S1). The ranges of lipids, apoB, and CRP measured in our sample (see Supplemental Material, Table S2) were comparable to reference levels for pregnant women in other populations (Klajnbard et al. 2010; Larsson et al. 2008).

Compared with unexposed women, those exposed to tobacco smoke *in utero* had higher median levels of triglycerides and CRP, and lower mean HDL (Table 2). After adjusting for age, physical activity, and education, significant associations with triglycerides [13.4% higher; 95% confidence interval (CI): 6.4, 20.9] and HDL (2.6 mg/dL lower; 95% CI: –5.0, –0.2) remained. Associations with all outcomes were similar after additional adjustment for personal smoking, but were attenuated after adjusting for BMI (Table 3). Average values of LDL, total cholesterol, and apoB tended to be higher among women exposed to tobacco smoke *in utero*, but the estimates were imprecise. The positive association of CRP with exposure to tobacco smoke *in utero* was still evident (though nonsignificant) after adjusting for age, physical activity, and education (16.3% higher in the exposed vs. unexposed women; 95% CI: –2.6, 39.0), but was close to the null after adjusting for BMI (2.7% higher; 95% CI: –12.7, 20.9) (Table 3). When we analyzed the data from all women using weighted linear regression, *in utero* tobacco smoke exposure remained associated with triglycerides (12.1% higher in the exposed women; 95% CI: 5.5, 19.2), HDL (2.3 mg/dL lower; 95% CI: –4.5, –0.1), and CRP (14.7% higher; 95% CI: –1.4, 33.5); estimates for LDL (3.3 mg/dL higher; 95% CI: –2.5, 9.1), total cholesterol (2.3 mg/dL higher; 95% CI: –4.5, 9.1), and apoB (2.9 mg/dL higher; 95% CI: –1.5, 7.2) were similar to those from the base sample. After adjusting for BMI, the estimates were again attenuated, but were consistent with those from the base sample; *in utero* exposure to tobacco smoke remained associated with triglycerides (9.4% higher in the exposed vs. unexposed women; 95% CI: 3.1, 16.1) and HDL (1.7 mg/dL lower; 95% CI: –3.9, 0.6), but not with CRP (2.1% higher; 95% CI: –10.9, 17.1).

**Table 2:** Plasma lipids and apoB (mg/dL) and CRP (mg/L) by *in utero* exposure to tobacco smoke (no/yes) among women from the base sample at enrollment (*n* = 479).

Plasma level	No (*n* = 345)	Yes (*n* = 134)	*p*-Value^a^
Triglycerides	117.2 (47.0)	135.7 (71.0)	< 0.01
HDL	68.4 ± 11.6	65.3 ± 12.7	0.01
LDL	126.5 ± 29.4	130.4 ± 32.4	0.20
Total cholesterol	212.8 ± 33.7	215.2 ± 38.5	0.50
ApoB	99.7 ± 21.7	103.4 ± 24.5	0.11
CRP (mg/L)	4.0 (4.5)	5.0 (5.4)	0.01
Plasma levels are presented as means ± SD or geometric means (interquartile range). ap-Values are from unadjusted linear regressions, except for triglycerides and CRP; two-sample Wilcoxon rank–sum (Mann–Whitney U) test for equality-of-medians was used for triglycerides and CRP.						

**Table 3:** Estimated associations [βs (95% CIs)] of *in utero* exposure to tobacco smoke with lipids, apoB, and CRP among adult women from the base sample (*n* = 479).

Outcome	Unadjusted		Adjusted										
			Age, physical activity, and education only		Plus personal smoking		Plus BMI		Plus personal smoking and BMI	
Triglycerides	15.8	(8.7, 23.4)	13.4	(6.4, 20.9)	13.4	(6.4, 21.0)	10.6	(3.8, 17.8)	10.7	(3.9, 17.9)
HDL	–3.2	(–5.5, –0.8)	–2.6	(–5.0, –0.2)	–2.5	(–4.9, –0.1)	–2.0	(–4.4, 0.4)	–1.9	(–4.3, 0.5)
LDL	3.9	(–2.1, 10.0)	3.3	(–2.9, 9.4)	3.2	(–3.0, 9.4)	2.8	(–3.4, 9.1)	2.8	(–3.5, 9.1)
Total cholesterol	2.4	(–4.6, 9.4)	2.1	(–5.1, 9.2)	2.3	(–4.9, 9.4)	1.9	(–5.3, 9.2)	2.1	(–5.2, 9.3)
ApoB	3.7	(–0.8, 8.2)	2.8	(–1.7, 7.4)	2.9	(–1.7, 7.5)	2.2	(–2.4, 6.8)	2.3	(–2.4, 6.9)
CRP	24.6	(4.5, 48.5)	16.3	(–2.6, 39.0)	15.9	(–3.0, 38.5)	2.7	(–12.7, 20.9)	2.9	(–12.6, 21.1)
Associations are expressed as the percent difference in the geometric mean for logn-transformed outcomes (mg/dL triglycerides and mg/L CRP) or the difference in mean values (mg/dL of HDL, LDL, total cholesterol, and ApoB) in the exposed compared with the unexposed group.															

Among 397 women in the base sample with known birth weight (Table 4), triglycerides remained higher and HDL lower among exposed compared with unexposed women before and after adjusting for birth weight (in addition to age, education, BMI, personal smoking, and physical activity), and when the analysis was restricted to 381 women born at term (i.e., gestational age ≥ 37 weeks). Other lipids, apoB, and CRP were again unrelated to *in utero* exposure to tobacco smoke (Table 4). Associations were similar after adjusting for birth weight among all women (data not shown).

**Table 4:** Adjusted^a^ coefficients [βs (95% CIs)] for lipids, apoB, and CRP by *in utero* exposure to tobacco smoke among women from the MoBa base sample with available birth weight.

Outcome	All women with available birth weight (*n* = 397)					Women born at term (*n* = 381)^b^	
	Before adjusting for birth weight		Adjusted for birth weight^c^		Adjusted for birth weight	
Triglycerides	11.1	(3.6, 19.1)	9.5	(2.1, 17.4)	10.2	(2.6, 18.5)
HDL	–3.2	(–5.9, –0.6)	–3.6	(–6.3, –0.9)	–4.2	(–7.0, –1.4)
LDL	0.9	(–5.6, 7.5)	0.0	(–6.6, 6.6)	–1.8	(–8.6, 5.1)
Total cholesterol	–0.7	(–8.3, 6.8)	–2.1	(–9.7, 5.5)	–3.6	(–11.5, 4.3)
ApoB	1.0	(–3.8, 5.8)	0.5	(–4.3, 5.4)	–0.2	(–5.2, 4.8)
CRP	5.0	(–12.3, 25.7)	3.4	(–13.9, 24.1)	0.4	(–16.7, 21.0)
Associations are expressed as the percent difference in the geometric mean for logn-transformed outcomes (mg/dL triglycerides and mg/L CRP) or the difference in mean values (mg/dL of HDL, LDL, total cholesterol, and ApoB) in the exposed compared with the unexposed group. aAll models were adjusted for age, education, physical activity, smoking, and BMI at blood draw. bGestational age ≥ 37 weeks. cAdditionally adjusted for participant’s gestational age at birth.									

Compared with unexposed women, women exposed to tobacco smoke *in utero* were more likely to have triglycerides ≥ 200 mg/dL [adjusted OR (aOR) = 2.8; 95% CI: 1.4, 5.7], HDL < 50 mg/dL (aOR = 2.4; 95% CI: 1.1, 5.1), and CRP ≥ 8 mg/L (aOR = 1.6; 95% CI: 1.0, 2.6); the odds of having cholesterol ≥ 240 mg/dL was slightly higher (aOR = 1.4, 95% CI: 0.9, 2.3) among exposed women (Table 5). After additional adjustment for BMI, the association of *in utero* exposure to tobacco with high triglycerides and low HDL remained statistically significant, but that was not the case for CRP. In the analysis that included all women, the associations were consistent with those from the base sample (Table 5).

**Table 5:** Adjusted^a^ ORs (95% CIs) for selected lipids and CRP by *in utero* exposure to tobacco smoke among MoBa participants.

Outcome	Base sample (*n* = 479)							All women (*n* = 823)^b^						
	Before adjusting for BMI		Adjusted for BMI		Before adjusting for BMI		Adjusted for BMI	
									*n*^c^	*n*^c^
Triglycerides ≥ 200 mg/dL	38	2.8	(1.4, 5.7)	2.5	(1.3, 5.1)	72	2.5	(1.3, 4.7)	2.2	(1.2, 4.3)
HDL < 50 mg/dL	33	2.4	(1.1, 5.1)	2.3	(1.1, 5.0)	61	2.1	(1.1, 4.0)	2.0	(1.0, 4.1)
Total cholesterol ≥ 240 mg/dL	97	1.4	(0.9, 2.3)	1.4	(0.8, 2.3)	159	1.4	(0.9, 2.2)	1.4	(0.9, 2.2)
CRP ≥ 8 mg/L	106	1.6	(1.0, 2.6)	1.2	(0.7, 2.1)	197	1.6	(1.1, 2.5)	1.2	(0.8, 2.0)
aAll models were adjusted for age, education, physical activity, and smoking. bLogistic regression models weighted for sampling probability. cNumber of participants classified as having the outcome.																

Compared with women who were unexposed and had a BMI of < 25 (kg/m^2^), women exposed to tobacco smoke *in utero* tended to have higher odds of high triglycerides, low HDL, and high total cholesterol regardless of their BMI (< 25 or ≥ 25), whereas women with BMI ≥ 25 tended to have higher odds of high CRP regardless of their exposure; the confidence intervals, however, were wide (Table 6). Among unexposed women, higher BMI (≥ 25 kg/m^2^) was also associated with higher odds of having high triglycerides, low HDL, and high total cholesterol.

**Table 6:** Adjusted^a^ ORs (95% CIs) for selected clinical chemistries by *in utero* exposure to tobacco smoke and BMI (< 25 and ≥ 25 kg/m2) among adult women.

*In utero* exposure to tobacco smoke/BMI	*n*	Triglycerides ≥ 200 mg/dL		HDL < 50 mg/dL		Total cholesterol ≥ 240 mg/dL		CRP ≥ 8 mg/L	
Base sample	479
Unexposed/BMI < 25	195	1.0	1.0	1.0	1.0
Unexposed/BMI ≥ 25	150	3.5	(1.2, 10.3)	2.5	(0.9, 6.9)	1.5	(0.9, 2.7)	4.0	(2.2, 7.1)
Exposed/BMI < 25	58	4.7	(1.4, 15.7)	4.4	(1.4, 14.4)	1.7	(0.8, 3.5)	1.1	(0.4, 2.8)
Exposed/BMI ≥ 25	76	6.9	(2.3, 20.6)	3.5	(1.2, 10.7)	1.7	(0.9, 3.4)	6.1	(3.2, 12.0)
All womenb	823
Unexposed BMI < 25	318	1.0	1.0	1.0	1.0
Unexposed BMI ≥ 25	265	3.1	(1.2, 7.8)	2.5	(1.0, 6.1)	1.5	(0.9, 2.4)	4.0	(2.4, 6.9)
Exposed BMI < 25	98	3.8	(1.4, 10.8)	3.8	(1.3, 11.1)	1.7	(0.9, 3.2)	1.2	(0.5, 2.6)
Exposed BMI ≥ 25	142	5.5	(2.1, 14.0)	3.2	(1.2, 8.2)	1.7	(0.9, 3.1)	6.1	(3.3, 11.2)
aAll models were adjusted for age, education, physical activity, and smoking. bLogistic regression models weighted for sampling probability.														

Estimates from models adjusted for participant’s age and physical activity only (the minimum sufficient adjustment set based on a DAG), fell between unadjusted estimates and estimates adjusted for age, education, and physical activity [triglycerides: 15.3% higher in the exposed vs. unexposed women (95% CI: 8.1, 22.9); HDL: 2.9 mg/dL lower (95% CI: –5.2, –0.5); and CRP: 20.8% higher (95% CI: 1.2, 44.1)]. The results from Tables 3 and 5 remained essentially the same after additional adjustment for gestational week at blood draw and income (data not shown). The results obtained from the multiple imputation analyses were comparable to those observed based on complete data (data not shown). Among women selected as cases (i.e., subfecund), *in utero* exposure to tobacco smoke was not associated with any outcome before or after adjusting for BMI; after BMI was added to the models, the signs of the estimates for triglycerides, HDL, and CRP were in the direction opposite to those shown before adjusting for BMI [see Supplemental Material, Table S3 (http://dx.doi.org/10.1289/ehp.1104563)].

## Discussion

In the present study, exposure to tobacco smoke *in utero* was associated with higher triglycerides and lower HDL during adulthood in a population of pregnant women. These adverse alterations in plasma lipids are compatible with the metabolic syndrome, and therefore of clinical relevance. The odds of having elevated triglycerides or low HDL levels were doubled among women exposed to tobacco smoke *in utero* as compared with unexposed women. Plasma LDL, total cholesterol, and apoB were not associated with the exposure in the adjusted analyses. Women exposed to tobacco smoke *in utero* also tended to have higher CRP, although this association was explained by the association of the exposure with BMI.

An adverse lipid profile early in life has been reported among children and adolescents exposed to tobacco smoke *in utero* as well as among those exposed to parental smoking during childhood (Huang et al. 2007; Metsios et al. 2011). Among 8,815 men and women (approximately 45 years of age) from the 1958 British birth cohort, *in utero* exposure to tobacco smoke was associated with high triglycerides and low HDL, although the latter was limited to women (Power et al. 2010). However, after accounting for life-time covariates and potential mediators (e.g., birth weight, gestational age, breast-feeding, education, physical activity, personal smoking) simultaneously in the analysis, these associations were weaker and no longer statistically significant (Power et al. 2010). Similarly, among 3,824 men and women (approximately 23 years of age) from a Brazilian birth cohort, *in utero* exposure to tobacco smoke was associated with low HDL in women but not in men; this association was also weakened and no longer statistically significant after accounting for confounders and potential mediators (e.g., birth weight, physical activity, personal smoking, BMI, waist circumference) (Horta et al. 2011). In the present study, the positive association between triglycerides and *in utero* exposure to tobacco smoke was consistent and remained after all adjustments; it was also supported by our analysis using the recommended clinical cut point used to define high triglycerides (≥ 200 mg/dL) (NCEP 2001). As reported in previous studies (Horta et al. 2011; Power et al. 2010), the association between reduced HDL and exposure to tobacco smoke *in utero* may not be independent of adult BMI; however, when using the recommended cut point for defining low HDL (for women, < 50 mg/dL) (NCEP 2001) the odds were doubled among exposed compared with unexposed women.

Our results differ from two recent studies where *in utero* exposure to tobacco smoke was associated with total cholesterol in adulthood (Jaddoe et al. 2008; Wen et al. 2010). We observed a positive association between *in utero* exposure to tobacco smoke and total cholesterol, but associations were weak and estimates were imprecise. Jaddoe et al. (2008) reported that the association of *in utero* smoking with total cholesterol was stronger among participants with moderate overweight compared to those with normal BMI. In our data, there was no significant departure from additive or multiplicative joint effects of BMI and *in utero* exposure to tobacco smoke. In addition, the odds of having high cholesterol among women exposed to tobacco smoke *in utero* were similar when the BMI was < 25 and ≥ 25 kg/m^2^.

Whether *in utero* exposure to tobacco smoke (or parental smoking during childhood as a surrogate of *in utero* exposure) is a risk factor for metabolic syndrome during adulthood remains unclear (Hunt et al. 2006; Power et al. 2010). We did not have data on blood pressure, fasting glucose, or central obesity, which are also components of the metabolic syndrome in addition to plasma triglycerides and HDL (Alberti et al. 2009). However, a higher prevalence of hypertension and obesity in relation to tobacco smoke *in utero* has been previously reported among 74,023 women enrolled in MoBa, which included some of the participants in the present analysis (Cupul-Uicab et al. 2012).

In animals, fetal exposure to nicotine at doses relevant to humans (i.e., serum cotinine levels of 136–300 ng/mL as found among moderate to heavy smokers) results in long-term metabolic alterations in adulthood that are consistent with components of the metabolic syndrome in humans (Bruin et al. 2010). In humans however, other less studied constituents of tobacco smoke such as carbon monoxide might also contribute to adverse lipid profiles (i.e., carbon monoxide is associated with fetal hypoxia, which can increase oxidative stress and potentially alter lipid metabolism in the fetus) (Chelchowska et al. 2011; Wen et al. 2010).

As in other observational studies, causal associations cannot be established with these data. It is possible that *in utero* exposure to tobacco smoke is acting as a marker of socioeconomic disadvantage during childhood, and the latter may be related to a higher risk for adult disease (Donovan and Susser 2011). Lifetime socioeconomic status of the participants was not available in MoBa. As shown in previous studies and in the present study, adjusting for factors such as BMI usually leads to weaker associations; however, the appropriateness of adjusting for risk factors that are affected by the exposure is debatable (Cole and Hernan 2002).

Estimated effects of exposure in conjunction with BMI showed strong associations of *in utero* exposure to tobacco smoke with high triglycerides (aOR = 4.7; 95% CI: 1.4, 15.7) and reduced HDL (aOR = 4.4; 95% CI: 1.4, 14.4) among women with normal BMI (Table 6). The combined estimate was consistent with additive effects of BMI and exposure on triglycerides and HDL, but the statistical power of the study for evaluation of interaction was limited. High BMI was associated with high CRP regardless of exposure, and the combined estimate suggests a weak association between *in utero* exposure to tobacco smoke and high CRP among overweight or obese women.

Although exposure data were collected retrospectively, reported exposure to maternal tobacco smoke *in utero* by the adult offspring has been shown to be valid (Simard et al. 2008); and previous analyses from MoBa participants also supports the validity and reproducibility of self-reported exposure to tobacco smoke *in utero* (Cupul-Uicab et al. 2011a, 2011b). An analysis of data from multiple populations indicated that birth weight is 149 g lower, on average, in children whose mothers smoked during pregnancy compared with children whose mothers did not smoke (Kramer 1987). The estimated average 181-g reduction in birth weight associated with *in utero* exposure to tobacco smoke in a subset of 11,082 MoBa participants indirectly supports the validity of self-reported *in utero* exposure (Cupul-Uicab et al. 2011b). The reproducibility of self-reported *in utero* exposure to tobacco smoke among MoBa participants who completed questionnaires for multiple pregnancies was high (weighted κ = 0.80) (Cupul-Uicab et al. 2011b). The intensity of *in utero* exposure to tobacco smoke was not ascertained in MoBa; therefore we were unable to assess a dose–response relationship.

The prevalence of *in utero* exposure to tobacco smoke in the present study (28% in the base sample) was similar to that for the MoBa cohort as a whole (approximately 27.8%), but the association between *in utero* exposure to tobacco smoke and obesity was slightly stronger in the base sample (aOR = 2.0; 95% CI: 1.10, 3.77) than in the full MoBa cohort (aOR = 1.53; 95% CI: 1.45, 1.61) (Cupul-Uicab et al. 2012). As expected, physical activity was associated with higher HDL and lower triglycerides, cholesterol, and LDL in the present study (data not shown).

Among subfecund women (i.e., with a time to pregnancy > 12 months), *in utero* exposure to tobacco smoke was not associated with lipid, apoB, or CRP levels. Subfecund women are more likely to have conditions associated with lipid alterations (e.g., polycystic ovary syndrome) (Norman et al. 2007) that are probably stronger predictors of the outcomes than *in utero* exposure to tobacco smoke, which may explain the lack of association among this subset of women.

In the present study, lipids were measured in nonfasting plasma specimens. Although triglycerides are known to be higher in the postprandial period, a recent prospective study among 26,509 women suggests that nonfasting triglycerides may be a better predictor of cardiovascular events (Bansal et al. 2007). The impact of adjusting for recent food consumption on associations between *in utero* exposure to tobacco smoke and components of the metabolic syndrome was negligible in the 1958 British cohort, a previous study that also measured lipids in nonfasting samples (Power et al. 2010). Because the cutoff points to define high triglycerides are based on fasting levels of triglycerides, potential misclassification of this outcome may be a concern; however, this might not be a concern for our analysis based on the continuous outcome. Furthermore, our results may not be directly comparable to those from fasting subjects or from non-pregnant women, and they may not be generalizable to non-pregnant women. Whether or not metabolic syndrome is a real entity (Kahn et al. 2005), clearly hypertriglyceridemia is an independent risk factor for coronary heart disease (NCEP 2001).

## Conclusion

Women exposed to tobacco smoke *in utero* were more likely to have high triglycerides and low HDL as adults. These adverse alterations in plasma lipids are compatible with metabolic syndrome and may have implications for future cardiovascular disease among the exposed.

## Supplemental Material

(209 KB) PDF
