# Supplementary material for: Exposure to Tobacco Smoke in Utero and Subsequent Plasma Lipids, ApoB, and CRP among Adult Women in the MoBa Cohort
Source: Environ Health Perspect. 2012 Jul 19;120(11):1532–7. doi: 10.1289/ehp.1104563 (PMC3556606; doi:10.1289/ehp.1104563)
Supplement: (209 KB) PDF [file ehp.1104563.s001.pdf]

Supplemental Material

Exposure to Tobacco Smoke *in Utero* and Subsequent Plasma Lipids, ApoB and CRP Among Adult Women in the MoBa Cohort

Lea A. Cupul-Uicab[1]*, Rolv Skjaerven[2,3], Kjell Haug[2], Gregory S. Travlos[4], Ralph E. Wilson[4], Merete Eggesbø[5], Jane A. Hoppin[1], Kristina W. Whitworth[1], Matthew P. Longnecker[1]

[1]Epidemiology Branch, National Institute of Environmental Health Sciences, NIH/DHHS/USA, Research Triangle Park, NC, USA

[2]Department of Public Health and Primary Health Care, University of Bergen, Bergen, Norway

[3]Medical Birth Registry of Norway, the Norwegian Institute of Public Health, Bergen, Norway

[4] Cellular and Molecular Pathology Branch, National Institute of Environmental Health Sciences, NIH/DHHS/USA, Research Triangle Park, NC, USA

[5]Department of Genes and Environment, Division of Epidemiology, Norwegian Institute of Public Health, Oslo, Norway

*Corresponding author:

Lea A. Cupul-Uicab, ScD.

National Institute of Environmental Health Sciences, MD A3-05, 111 TW Alexander Dr, Research Triangle Park, North Carolina 27709, USA; e-mail: cupuluicabl@niehs.nih.gov, acuicab@hotmail.com; tel. 919 541-3630; fax 919 541-2511

Supplemental Material, Table S1. Characteristics of women from the MoBa cohort according to their selection status

Characteristics	Selection status: Base sample (n = 479) %	Selection status: Subfecund women[a] (n = 344) %	p[b]
Age (years)			< 0.01
< 25	9.0	7.6	
25 - 29	38.2	26.7	
30 - 34	37.4	46.5	
≥ 35	15.4	19.2	
Education			< 0.01
< High School	6.7	12.5	
High School	29.4	34.9	
College	44.7	38.1	
> College	19.2	14.5	
Individual annual income (USD)			0.37
< 30,847	24.8	29.7	
30,847 - 46,269	41.8	36.6	
46,270 - 61,693	24.0	24.7	
> 61,693	9.4	9.0	
Parity			< 0.01
0	43.2	57.6	
1	38.8	29.9	
≥ 2	18.0	12.5	
BMI (kg/m^2)[c]			0.01
< 25.0	52.8	47.4	
25.0 - < 30.0	33.6	31.1	
30.0 +	13.6	21.5	

Supplemental Material, Table S1 (cont.)

Characteristics	Selection status: Base sample (n = 479) %	Subfecund women[a] (n = 344) %	p[b]
Smoking (cigarettes/day)[c]			0.01
Non smoker	78.9	72.7	
< 10	18.2	20.1	
10 +	2.9	7.3	
Alcohol drinking[c]			0.16
Non drinker	75.6	81.1	
Yes	15.2	11.3	
Missing	9.2	7.6	
Physical activity (times/week)[c]			0.73
None	20.7	22.7	
< 4.0	54.5	54.4	
4.0 - < 7.5	18.6	18.3	
≥ 7.5	6.3	4.7	
Exposed to tobacco smoke *in utero*	28.0	30.8	0.38
Triglycerides ≥ 200 mg/dL	7.9	9.9	0.33
HDL-C < 50 mg/dL	6.9	8.1	0.50
Total Cholesterol ≥ 240 mg/dL	20.3	18.0	0.43
CRP ≥ 8 mg/L	22.1	26.5	0.15

[a] Women with a time-to-pregnancy > 12 months

[b] p-values are from Pearson's chi-squared test comparing the base sample and subfecund women

[c] Reflects the woman's status at blood draw

Supplemental Material, Table S2. Distribution of plasma lipids, apoliprotein B, and CRP among women from the base sample (n=479)

Plasma levels (mg/dL)	Mean ± SD	Selected percentiles			
		25th	50th	75th	95th
Triglycerides	128.8 ± 44.1	96.0	121.0	152.0	213.0
HDL-C	67.5 ± 12.0	59.0	68.0	75.0	88.0
LDL-C	127.6 ± 30.3	107.0	125.0	147.0	180.0
Total Cholesterol	213.4 ± 35.1	189.0	211.0	234.0	280.0
ApoB	100.7 ± 22.6	85.0	99.0	114.0	143.0
CRP (mg/L)	6.4 ± 8.2	2.6	4.0	7.4	17.7

Supplemental Material, Table S3. Estimated associations of *in utero* exposure to tobacco smoke with lipids, apolipoprotein B, and CRP among subfecund[a] women (n=344)

			Adjusted							
	Unadjusted		Age, education, and physical activity only		Plus personal smoking		Plus BMI		Plus personal smoking and BMI	
Outcomes[b]	β	95% CI	β	95% CI	β	95% CI	β	95% CI	β	95% CI
Triglycerides	2.3	(-5.1, 10.3)	1.0	(-6.4, 9.0)	0.3	(-7.1, 8.3)	-1.6	(-8.7, 5.9)	-2.3	(-9.3, 5.3)
HDL-C	-1.0	(-3.8, 1.8)	-0.5	(-3.3, 2.3)	-0.5	(-3.3, 2.4)	0.3	(-2.5, 3.1)	0.3	(-2.5, 3.1)
LDL-C	1.9	(-5.0, 8.7)	2.6	(-4.4, 9.5)	2.7	(-4.3, 9.7)	1.6	(-5.4, 8.6)	1.7	(-5.3, 8.7)
Total cholesterol	0.5	(-7.4, 8.4)	1.6	(-6.4, 9.6)	1.5	(-6.5, 9.6)	1.5	(-6.6, 9.5)	1.4	(-6.7, 9.6)
ApoB	1.4	(-3.8, 6.6)	1.7	(-3.6, 6.9)	1.5	(-3.8, 6.9)	0.6	(-4.7, 5.9)	0.5	(-4.8, 5.8)
CRP	4.7	(-13.4, 26.6)	1.9	(-15.9, 23.5)	2.4	(-15.6, 24.4)	-7.8	(-22.7, 10.0)	-7.1	(-22.2, 11.0)

[a] Women with a time-to-pregnancy > 12 months

[b] Associations are expressed as the % difference in the geometric mean for $\log_n$-transformed outcomes (mg/dL triglycerides and mg/L CRP) or the difference in mean values (mg/dL of HDL-C, LDL-C, total cholesterol, and apoB) in the exposed compared with the unexposed group.

Supplemental Material, Figure S1. Directed acyclic graph for the selection of confounders when assessing the association of *in utero* exposure to tobacco smoke with plasma lipids, apolipoprotein B, and CRP among adult women from the MoBa cohort

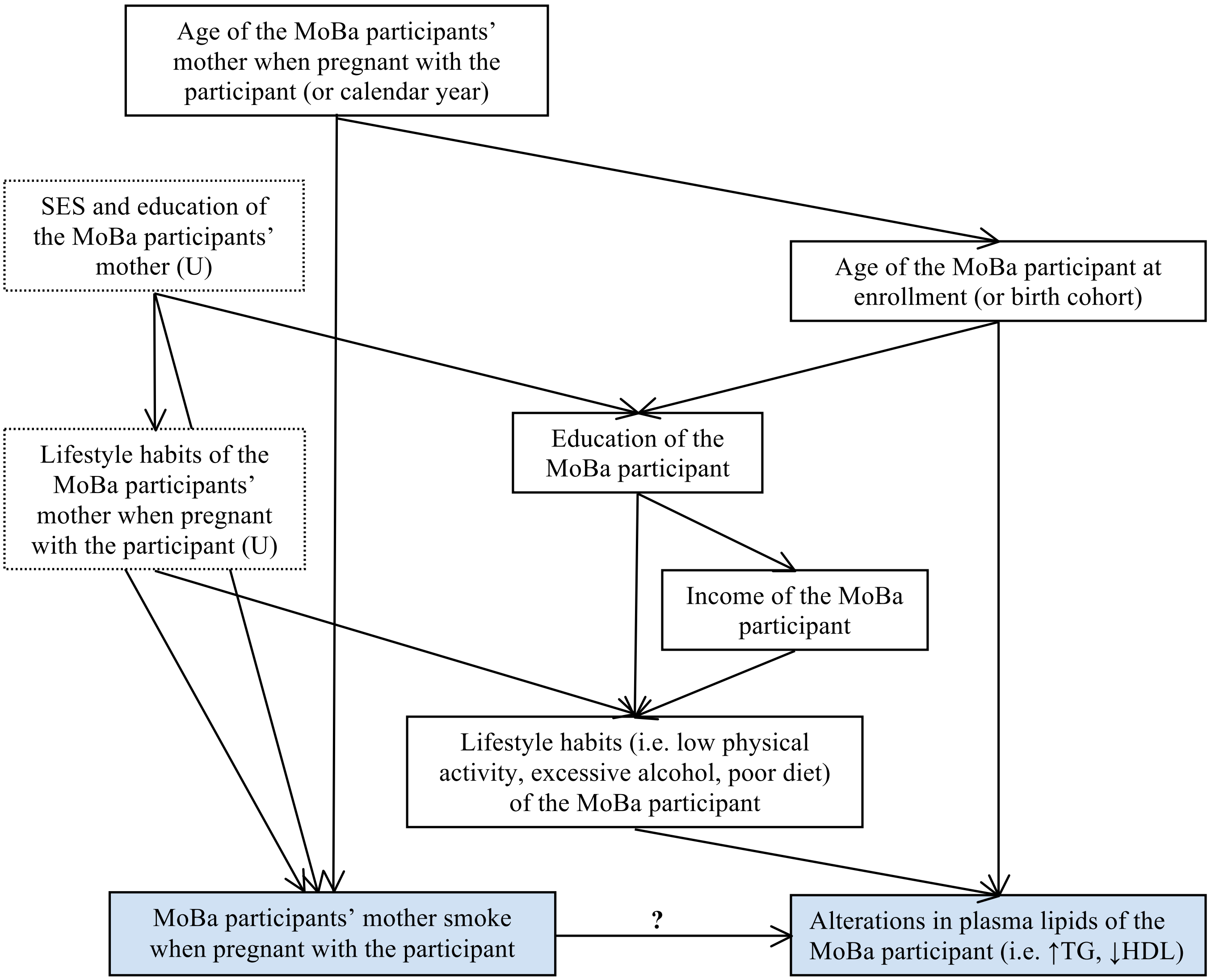

Variables connected by an arrow with a question mark, represents the association of interest. Variables from the mother (in dotted rectangles) were potential confounders that were unmeasured (U). This DAG represents an oversimplification of the potential association of *in utero* exposure to tobacco smoke with the outcomes.
